# The Supply of Medical Students

**Published:** 1915-10-09

**Authors:** 


### The Hospital

The Workers' Newspaper of Administrative Medicine and Institutional Life, Administration, National Insurance and Health.

No. 1530, Vol. LIX. SATURDAY, OCTOBER 9, 1915. PRICE ONE PENNY.

### THE SUPPLY OF MEDICAL STUDENTS.

At the moment the special claim of the nation on the medical profession is for the supply of fully-qualified doctors ready and willing to take service with the new armies which are being sent into the field. The satisfaction of this claim is, of course, causing acute difficulties in hospital and civil practice, but of these it is useless to complain. The demands to which all others must yield are the successful prosecution of the war and the efficient medical and surgical care of our sick and wounded soldiers. Hence, both doctors who remain at home and patients who look to them for help must make the best of circumstances, and must try to comfort themselves with the reflection that in this way they are helping the common cause. The situation is an unexpected one, for even the wisest of the prophets could not have anticipated so vast a dislocation of medical work. But while all eyes are turned anxiously on the immediate position, it is well that some thought also should be given to the future. The services demanded of the medical profession will not cease with the termination of the war, and therefore it is necessary to consider whether adequate provision is being made for the return of peace. Just now it is the cry of the Army for doctors which is heard, and other claims in the same direction receive little attention. But the time will come when normal conditions will assert themselves, when the public will put in its demand for attention, and the profession will have to justify its position in the body politic. For the medical practitioner of the future it is necessary to secure the medical student of the present, and failure at the moment will reveal itself in a serious fashion some five or six years hence.

There is, of course, no doubt that young men who otherwise would have commenced the study of medicine have been diverted to the Army, and a considerable number of those who have made some progress with their studies have left these for the fighting ranks. It is, therefore, certain that during the next few years there will be a diminished production of qualified practitioners. This is inevitable in the circumstances, and no ingenuity can neutralise such a result. Quite recently the War Office have declined to advise junior students of medicine to continue their studies rather than to elect for active service, and in view of this, medical authorities, whatever be their private opinions, can hardly urge an opposite course. Doubtless it is legislation for the moment rather than for the future, but the moment is a vital one and everything must yield to its demands. If the Army needs men it must have them, even though a year or two hence the British public finds itself short of doctors.

There is, however, a more remote view to be taken. What is to be the position some five or six years hence? The answer to this question depends on the number of students who enter the medical schools during the present session, and the returns on this point will be anxiously expected. For the moment the promise is not a good one, but we have reason to believe that it will not be so defective as some of the pessimists have anticipated. Of course, many lads who are too young to enter the Army are quite old enough to begin medical study, and the efforts which have been made to bring the position clearly before parents and their sons are not likely entirely to have failed. Amidst the excitement and turmoil of the national situation the importance of the medical profession has been set in a strong light, and this can hardly have missed the eye of ambitious and adventurous youth. We are, therefore, little disposed to despair of the outlook, and though there will certainly be a period during which medical practitioners will be a short supply, the inherent attractions of medicine will, we doubt not, gradually re-assert themselves.

Concerning the various expedients which have been proposed to meet the shortage of medical students there is nothing very hopeful to be said. To shorten the curriculum is a vain proposal, and leaves crying in the wilderness the few voices which urge it. Equally impossible, and perhaps still more disastrous, is the suggestion that the standard of examinations should be lowered. Whatever else the future may want, it will certainly not want imperfectly trained medical practitioners. More sympathy may be felt for the proposal to substitute for examinations certificates from teachers guaranteeing both the satisfaction of the curriculum and the attainment of efficiency. No doubt the examination fetish is overworshipped, but it cannot be deprived of its pride of place until some effective substitute is found, and here, in spite of effort, human ingenuity has hitherto run aground. Moreover, none of these expedients, except in a very indirect fashion, would help to increase the number of students. They might make the path easier and the course more rapid, but they would not in themselves do much to multiply disciples. One hindrance to the medical curriculum is doubtless the expense, and with modern laboratory demands and the like there is no chance that this can be lessened. A suggestion to try to meet this is that a fund should be raised to provide medical education for the sons of doctors who have fallen in the war. Even if this pleasing proposal were adopted the increase would hardly be appreciable, and in these days of funds a new one would have a hard struggle for existence. Doubtless a serious shortage of medical practitioners might compel State intervention, but the recognition of this possibility will not help the present situation. The best prospect we believe to be a reiterated presentation of the values of the profession and a courageous statement of its future prospects.

